# Antiplatelet-Proton Pump Inhibitor Interactions and Arterial Thrombotic Events: A Pharmacovigilance Assessment using Disproportionality and Interaction Analysis

**DOI:** 10.2174/011573403X374906250417043907

**Published:** 2025-05-12

**Authors:** Kannan Sridharan

**Affiliations:** 1 Department of Pharmacology & Therapeutics, College of Medicine & Health Sciences, Arabian Gulf University, Manama, Kingdom of Bahrain

**Keywords:** Aspirin, clopidogrel, lansoprazole, rabeprazole, omeprazole, antiplatelet therapy

## Abstract

**Introduction:**

The concomitant use of PPIs with antiplatelet therapy remains controversial due to potential drug interactions affecting clinical outcomes. While PPIs are recommended for gastroprotection in patients receiving antiplatelet therapy, concerns persist regarding their impact on antiplatelet efficacy, particularly with dual antiplatelet therapy (DAPT).

**Aims:**

The aim of this study is to evaluate the safety profiles of antiplatelet-proton pump inhibitors (PPIs) combinations and assess the clinical implications of their concurrent use in real-world settings through pharmacovigilance data analysis.

**Objectives:**

The objective of this study is to analyze and compare the thrombo-embolic risk profiles of various antiplatelet-PPI combinations using the FDA Adverse Event Reporting System database.

**Methods:**

We conducted a comprehensive analysis of the FDA Adverse Event Reporting System (FAERS) database to evaluate the thrombo-embolic risk associated with antiplatelet-PPI combinations. The reporting odds ratio (ROR) and information component were calculated to detect safety signals. The interaction signal score (INTSS) was used to assess the protective or harmful effects of adding acetylsalicylic acid to clopidogrel-PPI combinations.

**Results and Discussion:**

Analysis revealed significant safety signals for thrombo-embolic events with clopidogrel-rabeprazole (ROR: 62.67, 95% CI: 38.38-102.32) and clopidogrel-omeprazole (ROR: 6.87, 95% CI: 4.89-9.66) combinations. DAPT-PPI combinations showed comparable safety profiles to monotherapy-PPI combinations. The INTSS analysis suggested a potential protective effect of acetylsalicylic acid when added to clopidogrel-PPI combinations. Gender-specific analysis revealed female predominance in monotherapy complications and male predominance in combination therapy events. Clinical outcomes, including mortality and hospitalization rates, were comparable between groups.

**Conclusion:**

This pharmacovigilance analysis suggests that while DAPT-PPI combinations demonstrate acceptable safety profiles, careful consideration should be given to PPI selection, particularly given the unexpected safety signals with rabeprazole and confirmed risks with omeprazole. The addition of acetylsalicylic acid to clopidogrel-PPI combinations may offer protective effects against thrombo-embolic events. These findings support individualized risk-benefit assessment in selecting antiplatelet-PPI combinations while ensuring adequate gastroprotection for high-risk patients.

## 1 INTRODUCTION

Antiplatelet therapy, particularly with acetylsalicylic acid and clopidogrel, remains the cornerstone of thrombo-embolic event prevention in high-risk patients [1]. The concomitant use of proton pump inhibitors (PPIs) has become standard practice for patients receiving antiplatelet therapy with risk factors for gastrointestinal complications. These risk factors include advanced age (especially >75 years), history of gastroduodenal ulcers, gastrointestinal bleeding or perforation, *Helicobacter pylori* infection, and concurrent use of medications that enhance gastrointestinal bleeding risk [2].

The clinical implications of PPI co-administration with antiplatelet therapy, however, have yielded conflicting evidence in real-world studies. A comprehensive systematic review and meta-analysis demonstrated that while PPI-clopidogrel combinations were associated with increased risks of major adverse cardiac events and myocardial infarction in observational studies, these associations were not confirmed in randomized clinical trials. However, cardiovascular mortality risk remained unchanged [3].

Recent investigations have revealed nuanced interactions between specific PPIs and antiplatelet agents. A study focusing on Asian populations receiving clopidogrel therapy found no significant differences in platelet inhibition or responsiveness between PPI and non-PPI groups, with the notable exception of dexlansoprazole, which significantly impaired both platelet inhibition and clopidogrel responsiveness [4]. The interaction potential varies substantially among PPIs, with omeprazole demonstrating significant interference with clopidogrel's therapeutic efficacy, while pantoprazole shows minimal interaction [5]. Dual antiplatelet therapy (DAPT), combining acetylsalicylic acid and clopidogrel, has emerged as the standard of care for patients with coronary artery disease, acute coronary syndrome, and post-procedural management following percutaneous coronary intervention or coronary artery bypass grafting [6]. While PPIs are frequently co-prescribed with DAPT to minimize upper gastrointestinal bleeding risk [7], the interaction dynamics become more complex in this setting. Genetic factors add another layer of complexity to these drug interactions. Meta-analytic evidence indicates that clopidogrel-PPI combinations significantly increase major adverse cardiac outcomes (OR: 1.42; 95% CI: 1.30-1.55), with particularly pronounced effects in *CYP2C19* rapid metabolizers (*1/*1 genotype, OR: 1.42; 95% CI: 1.12-1.81) [8]. However, comprehensive data regarding PPI interactions, specifically with DAPT, remains limited. PPIs reduce gastric acid secretion, thereby increasing gastric pH that hinders the absorption of acetylsalicylic acid as more fractions of the administered drug become ionized [9]. Another study revealed that over a one-year follow-up period, 17% of patients (3,400 individuals) experienced a recurrence of myocardial infarction, stroke, or cardiovascular-related death with concomitant PPI and acetylsalicylic acid [10]. This study was a propensity-score matched analysis designed to adjust for baseline differences and potential confounding factors. The likelihood of the composite outcome (CV-related death, MI, and stroke) was significantly greater in patients using PPIs compared to non-users, with a hazard ratio (HR) of 1.6 Comparable findings were observed for secondary outcomes, including all-cause mortality (HR, 2.4), CV-related mortality (HR, 2.2), and MI (HR, 1.3) [10].

The United States Food and Drug Administration Adverse Event Reporting System (USFDA AERS) serves as a crucial pharmacovigilance tool, incorporating both mandatory manufacturer reports and voluntary healthcare provider submissions [11]. This database has proven invaluable in identifying drug safety signals and potential drug-drug interactions [12]. Given the existing knowledge gaps regarding PPI interactions with both antiplatelet monotherapy and DAPT, we conducted a comprehensive analysis of thrombo-embolic risk patterns using this extensive pharmacovigilance database. Our investigation specifically aimed to compare the risk profiles between antiplatelet monotherapy and dual antiplatelet therapy when combined with PPIs, addressing a critical need in current clinical practice.

## 2 MATERIALS AND METHODS

### 2.1 Data Source

This pharmacovigilance study analyzed data from the USFDA Adverse Event Reporting System (AERS) between Q1 2004 and Q3 2024. Thrombo-embolic events were identified using the Standardised MedDRA Query (SMQ) “Embolic and thrombotic events, arterial” (code: 20000082), with specific Preferred Terms detailed in Table **S1** [13].

### 2.2 Data Processing

The investigation targeted reports involving acetylsalicylic acid, clopidogrel, and their combinations with proton pump inhibitors (lansoprazole, dexlansoprazole, omeprazole, esomeprazole, pantoprazole, and rabeprazole). Reports containing anticoagulants were excluded to minimize confounding effects (Table **S2**). Deduplication followed USFDA guidelines, with case retention based on the most recent FDA_DT or Individual Safety Report numbers. For monotherapy analysis, only cases designating antiplatelets as “primary suspect” were included. Demographic parameters and reporting characteristics were documented for all cases.

### 2.3 Data Mining Algorithms

We employed a “case-non-case” disproportionality analysis methodology to evaluate the association between antiplatelets (alone and in combination with PPI) and arterial thrombo-embolic events [14]. The OpenVigil 2.1 platform was utilized for data extraction and analysis of antiplatelet-PPI-arterial thrombo-embolic event pairs. To ensure robust signal detection, we implemented both frequentist and Bayesian approaches, each offering complementary perspectives on safety signal detection.

The frequentist analysis incorporated the calculation of Reporting Odds Ratio (ROR), Proportional Reporting Ratio (PRR), and Relative Reporting Ratio (RRR). Signal detection adhered to Evans' criteria, requiring a minimum threshold of three reports, PRR >2, and chi-square (χ^2^) >4 for each drug-event pair [15]. We calculated 95% confidence intervals for both ROR and PRR, with signals confirmed when the lower limit of the ROR CI exceeded 1. The RRR provided additional context by comparing observed *versus* expected case frequencies within the database.

Bayesian methodologies included the Bayesian Confidence Propagation Neural Network (BCPNN) and Multi-item Gamma Poisson Shrinker (MGPS) analyses. The BCPNN utilized the Information Component (IC) metric, representing the logarithmic relationship between observed and expected co-occurrences. Signal detection was confirmed when the IC's lower 95% CI bound (IC025) exceeded zero. For MGPS, we employed the Empirical Bayes Geometric Mean (EBGM), with signals identified when the lower bound of the 95% CI (EBGM05) exceeded 2 [16].

### 2.4 Interaction Signal Scores

To assess potential drug interactions, we calculated Interaction Signal Scores (INTSS) between antiplatelet monotherapy and dual antiplatelet therapies with PPIs. The INTSS methodology incorporated EBGM scores with 90% confidence intervals (EB05 and EB95) for both monotherapy and combination therapy scenarios [17]. INTSS was calculated as follows (Eq. 1):


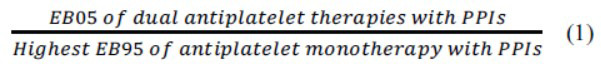


An INTSS exceeding 1 indicated a statistically significant drug interaction potentially increasing the risk of arterial thrombo-embolic risk [17].

### 2.5 Outcomes Assessed

We evaluated four primary clinical outcomes for all antiplatelet-PPI cases: mortality, life-threatening events, disability, and hospitalization (both initial admission and extended stays).

### 2.6 Compliance with Reporting Standards

This study adheres to the guidelines outlined in the REporting of A Disproportionality analysis for drUg Safety signal detection using spontaneously reported adverse events in Pharmacovigilance (READUS-PV) [18].

## 3 RESULTS

### 3.1 Search Results

**Fig. (1):**
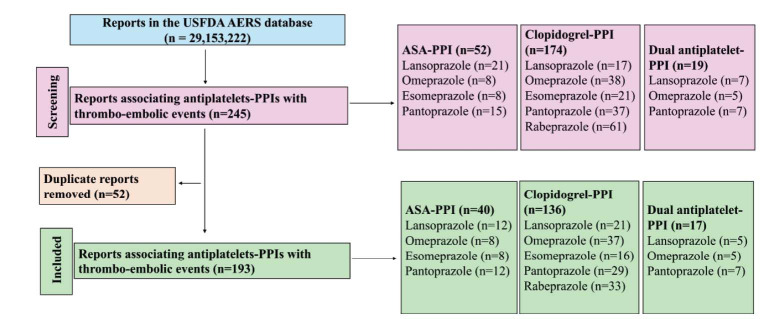
Study flow diagram. A total of 193 reports were included in this analysis. **Abbreviations**: PPI: Proton pump inhibitor; and ASA: Acetylsalicylic acid.

Analysis of the USFDA AERS database encompassed 29,163,222 reports, from which 193 reports met the predefined inclusion criteria. These comprised 176 reports related to antiplatelet monotherapy, and 17 reports related to dual antiplatelet therapies with PPIs (Fig. **1**). Only one report existed each for dexlansoprazole (in combination with clopidogrel) and esomeprazole and rabeprazole combinations with DAPT, and there were no reports for acetylsalicylic acid-rabeprazole combination. Demographic analysis, summarized in Table **1**, revealed that arterial thrombo-embolic events after antiplatelet-PPI potential interactions predominantly affected middle-aged and older adults (>40 years), with a female predominance observed in the monotherapy group, and male predominance in the antiplatelet combination group. Comparative analysis demonstrated that combination therapies were associated with a relatively higher risk of arterial thrombo-embolic events with clopidogrel with rabeprazole compared to other drugs, as illustrated in Fig. (**2**).

### 3.2 Signal Detection Measures for the Risk of Arterial Thrombo-Embolic Events with Antiplatelet-PPI Combinations

The comprehensive signal detection analysis for antiplatelet-PPI-arterial thrombo-embolic event risk is presented in Table **2**. Amongst the antiplatelet monotherapies with PPI, both frequentist and Bayesian signals were generated for acetylsalicylic acid combinations with esomeprazole, and clopidogrel combinations with omeprazole, esomeprazole, pantoprazole and rabeprazole. Amongst the DAPT-PPI combinations, lansoprazole and omeprazole were observed with frequentist and Bayesian signals.

**Fig. (4):**
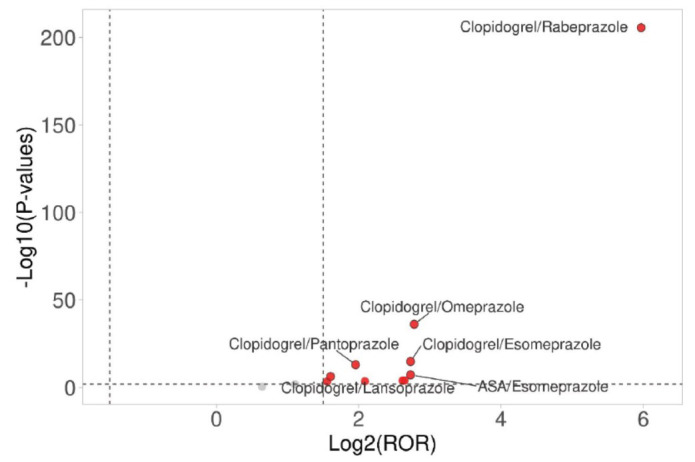
Volcano plot for antiplatelet-PPI combinations for the risk of arterial thrombo-embolic events. The red circles represent antiplatelet-PPI combinations and as farther they lie on both the x- and y-axes, more significant is the association of the drug with the risk of arterial thrombo-embolic events. The plot indicates that the most substantial arterial thrombo-embolic risk for the clopidogrel-PPI combinations, of which the highest risk was observed with rabeprazole followed by omeprazole combinations.

Fig. (**3**) presents ROR plots illustrating the relative reporting of thrombo-embolic events across different antiplatelet-PPI combinations. The analysis revealed that all antiplatelet-PPI combinations carried higher risks of thrombo-embolic events except the acetylsalicylic acid-omeprazole combination. In general, compared to clopidogrel-PPI combinations (except lansoprazole and pantoprazole), DAPT-PPI combinations were observed with relatively lower risks of thromboembolic events. These findings were further corroborated by volcano plot analysis (Fig. **4**), which demonstrated the most substantial arterial thrombo-embolic risk for the clopidogrel-PPI combinations, of which the highest risk was observed with rabeprazole followed by omeprazole combinations.

### 3.3 Measures of Interaction Signals

Table **3** presents the INTSS calculations for various antiplatelet-PPI combination therapies. Although no statistically significant drug interactions were identified for the dual antiplatelet-PPI combinations, the EBGM value of clopidogrel with omeprazole was higher than the dual antiplatelet-PPI combination, indicating a potential interaction of reduced thrombo-embolic events with concomitant acetylsalicylic acid in the DAPT with PPI.

### 3.4 Analysis of Reported Outcomes in Antiplatelet-PPI Combinations

The distribution of clinical outcomes between antiplatelet monotherapy- and DAPT-PPI combination therapy-associated arterial thrombo-embolic events is depicted in Fig. (**5**). No significant differences were observed in the outcomes between antiplatelet-PPI combinations (χ2: 3.5; df: 6; *p*: 0.7).

## 4 DISCUSSION

### 4.1 Key Findings

This pharmacovigilance analysis of the USFDA AERS database revealed several important findings regarding the safety profile of antiplatelet-PPI combinations.

- Our analysis identified significant safety signals for thrombo-embolic events with several antiplatelet-PPI combinations, particularly pronounced with clopidogrel-rabeprazole and clopidogrel-omeprazole combinations.
- While DAPT-PPIs generally showed lower risk profiles compared to clopidogrel-PPI monotherapy combinations, the INTSS analysis suggested a potential protective effect of acetylsalicylic acid when added to clopidogrel-PPI combinations.
- The demographic patterns revealed a gender-specific risk distribution, with female predominance in monotherapy complications and male predominance in combination therapy events.
- Despite these differential risk patterns, clinical outcomes, including mortality, hospitalization, and disability rates, were comparable between monotherapy and combination therapy groups. These findings provide important insights into the real-world safety implications of antiplatelet-PPI co-prescription and suggest the need for careful consideration of individual patient factors when selecting appropriate therapeutic combinations.

### 4.2 Comparison with Existing Literature

Our analysis revealed no significant differences in arterial thrombo-embolic event risk between DAPT-PPI combinations and monotherapy-PPI combinations, aligning with current evidence. This finding is supported by a comprehensive meta-analysis of eight studies comparing DAPT alone versus DAPT-PPI combinations, which found no significant difference in major adverse cardiac events (RR = 0.93, 95% CI = 0.81–1.06, *p* = 0.27) [19]. Furthermore, a recent meta-analysis encompassing six randomized trials (6,930 patients) and 16 observational studies (183,546 patients) demonstrated no significant differences in major adverse cardiac events, myocardial infarction, or all-cause mortality between DAPT-PPI and DAPT-non-PPI groups in randomized trials [20].

While observational studies have suggested potential associations between specific PPI subtypes (particularly lansoprazole and pantoprazole) and increased risks of major adverse cardiac events [20], our INTSS analysis did not demonstrate elevated arterial thrombo-embolic event risks with DAPT-PPI combinations (including lansoprazole, omeprazole, and pantoprazole) compared to clopidogrel monotherapy-PPI combinations.

A concerning trend in clinical practice is the significant under-prescription of PPIs due to interaction concerns. A revealing study of 400 acute coronary syndrome patients discharged on DAPT found that 54.5% had clear indications for GI prophylaxis, primarily due to advanced age (44.8%) and anticoagulant use (19.8%). Alarmingly, 86.7% of these patients were not prescribed PPIs, including 75.4% of those with multiple indications [21]. However, targeted quality improvement initiatives have demonstrated success in addressing this gap. One hospital project improved PPI prescription compliance from 56% to 100% through systematic interventions, including educational sessions for healthcare staff and implementation of pharmacy verification protocols [22]. Given our findings suggesting a potential protective effect of aspirin in DAPT-PPI combinations against thrombo-embolic events, similar systematic approaches to improve PPI prescription compliance with DAPT are warranted.

A notable finding from our analysis challenges current guidelines regarding specific PPI selection. While previous studies have demonstrated that omeprazole and esomeprazole competitively inhibit the *CYP2C19* pathway when combined with clopidogrel, leading to recommendations favoring rabeprazole or pantoprazole [23], our analysis revealed significantly increased reporting of arterial thrombo-embolic events with the clopidogrel-rabeprazole combination. This observation is supported by recent evidence in healthy volunteers showing rabeprazole's reduction of both maximum concentration and total exposure to active clopidogrel metabolite [24]. Additionally, in patients with cardiac conditions requiring intervention, rabeprazole significantly reduced platelet aggregation (81 ± 5%) [25]. However, this finding warrants careful interpretation, considering potential reporting bias and the multifactorial nature of thrombo-embolic events.

Among DAPT-PPI combinations, our analysis revealed the highest rate of arterial thrombo-embolic events with omeprazole, consistent with findings from a nationwide Taiwanese study. This study reported a significantly higher incidence rate of ischemic stroke in the omeprazole cohort (81.67 per 1000 person-years) compared to controls (57.45 per 1000 person-years), with an adjusted hazard ratio of 1.39 (95% CI 1.03–1.74) [26]. These findings suggest the need for careful selection of specific PPIs when prescribing in combination with antiplatelet therapy, particularly in high-risk patients.

### 4.3 Strengths, Limitations and Way Forward

The present study has several notable strengths, including its utilization of a large, real-world pharmacovigilance database, comprehensive analysis of multiple antiplatelet-PPI combinations, and robust statistical methodology incorporating both frequentist and Bayesian approaches. However, several limitations warrant consideration. First, the AERS database is subject to inherent reporting biases, including under-reporting, selective reporting, and the Weber effect. Second, the database lacks important clinical information such as dosing regimens, treatment duration, concurrent medications, and patient comorbidities, which could influence thrombo-embolic risk. Third, the absence of denominator data (total number of patients exposed to these drug combinations) limits the calculation of true incidence rates. Fourth, the causal relationship between drug combinations and adverse events cannot be definitively established due to the nature of spontaneous reporting systems. Fourth, despite adhering to the recommended deduplication process, residual duplicates may remain. Also, the absence of demographic details of those receiving antiplatelets with PPIs and not developing any thrombo-embolic events in the database precludes the application of any inferential statistical analysis. Future research directions should include: (1) large-scale prospective cohort studies with genetic profiling to evaluate the impact of *CYP2C19* polymorphisms on antiplatelet-PPI interactions, (2) randomized controlled trials comparing different PPI subtypes in combination with DAPT, particularly focusing on rabeprazole and newer PPIs, (3) development of risk prediction models incorporating patient-specific factors to guide PPI selection in antiplatelet therapy, and (4) real-world effectiveness studies of electronic alert systems and clinical decision support tools for optimizing antiplatelet-PPI prescribing patterns. Additionally, pharmacoeconomic analyses evaluating the cost-effectiveness of different antiplatelet-PPI combinations would provide valuable insights for clinical decision-making and healthcare policy.

## CONCLUSION

In conclusion, this large-scale pharmacovigilance analysis provides important insights into the safety profiles of antiplatelet-PPI combinations in real-world settings. Our findings suggest that while DAPT-PPI combinations demonstrate comparable safety profiles to monotherapy-PPI combinations, specific attention should be paid to the choice of PPI, particularly given the unexpected safety signals observed with rabeprazole-clopidogrel combinations and the confirmed risks with omeprazole. The addition of acetylsalicylic acid to clopidogrel-PPI combinations appears to offer potential protective effects against thrombo-embolic events, supporting current clinical practices of DAPT with appropriate PPI coverage. For clinical practice, these results indicate that pantoprazole and esomeprazole should be preferred when gastroprotection is needed in patients on DAPT, while omeprazole and rabeprazole should be avoided in clopidogrel-treated patients. The persistent under-prescription of PPIs in patients with clear indications for gastroprotection remains a concerning trend that needs to be addressed through systematic quality improvement initiatives. Clinicians should implement standardized protocols to identify high-risk patients requiring PPI prophylaxis, particularly those with a history of gastrointestinal bleeding, advanced age, or concurrent use of anticoagulants. These findings underscore the importance of individualized risk-benefit assessment when selecting antiplatelet-PPI combinations, considering patient-specific factors, comorbidities, and genetic variations. Moving forward, healthcare providers should focus on optimizing PPI selection in antiplatelet therapy while ensuring adequate gastroprotection for high-risk patients. Future prospective studies and randomized trials are needed to further elucidate the complex interactions between specific PPIs and antiplatelet agents, particularly in the context of dual antiplatelet therapy.

## Figures and Tables

**Fig. (2):**
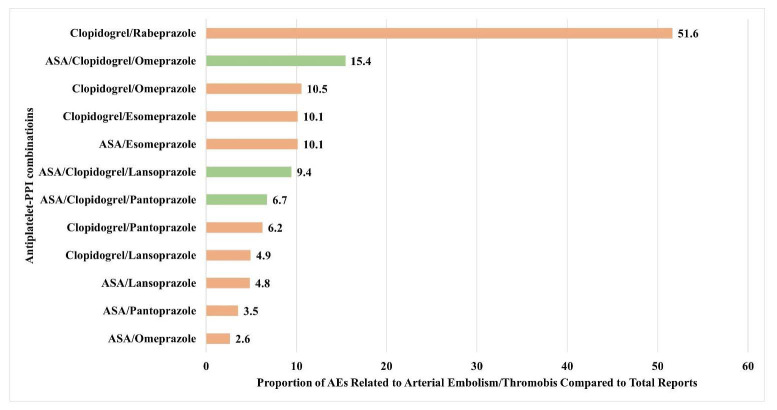
Comparison of rates of arterial thrombo-embolic events with antiplatelets. The horizontal bars represent the proportion of AEs reported as hypotension amongst the total AEs. Brown bars represent dual antiplatelet therapy, and green bars represent triple antiplatelet therapies. Clopidogrel and ASA/clopidogrel with PPIs were associated with higher rates of reporting arterial thrombo-embolic events compared to ASA with antiplatelets.

**Fig. (3):**
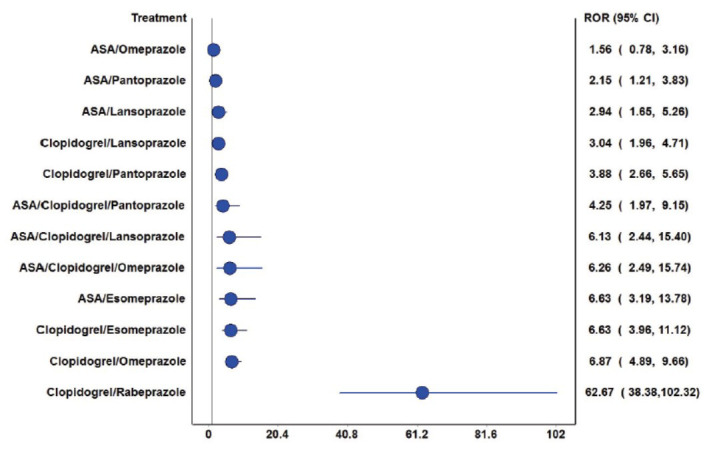
Comparison of RORs for the risk of arterial thrombo-embolic events. The blue circles represent the point estimates, and the horizontal lines represent the 95% CI of RORs. Vertical black line represents the line of no difference in the risk of arterial thrombo-embolic events. All antiplatelet-PPI combinations carried higher risks of thrombo-embolic events except acetylsalicylic acid-omeprazole combination.

**Fig. (5):**
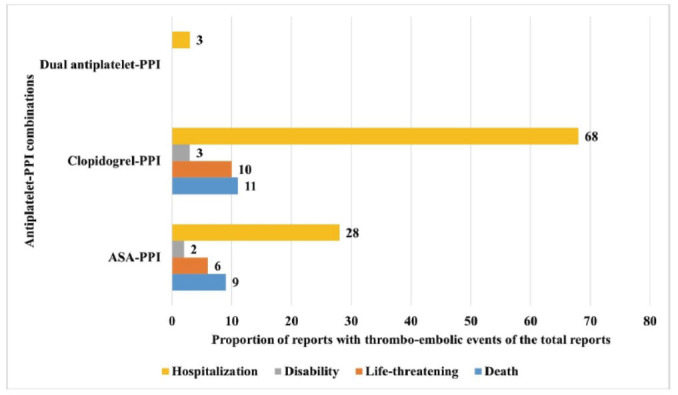
Comparison of reported outcomes in the reports associating antiplatelet-PPI combinations with arterial thrombo-embolic events. The horizontal bar chart depicts the distribution of key outcomes between the antiplatelet-PPI combinations. No significant differences were observed in the outcomes between antiplatelet-PPI combinations.

**Table 1:** Demographic characteristics of patients included in the reports.

**Characteristics**		**Antiplatelet Monotherapy-PPI Combinations**		**DAPT-PPI Therapy (n=17)**	
		**Acetylsalicylic Acid (n=40)**	**Clopidogrel (n=136)**		
3Age group [n (%)]	>40 to <65	13 (32.5)	24 (17.6)	1 (5.9)
	>65	18 (45)	65 (47.8)	13 (76.5)	
	Not specified	9 (22.5)	47 (34.6)	3 (17.7)	
Quantitative age (years)	Mean (SD)	68.1 (12.1)	71.7 (10.4)	74.7 (9.5)	
	Median (range)	73 (45-89)	69 (48-92)	79 (52-84)	
Gender [n (%)]	Male	19 (47.5)	48 (35.3)	11 (64.7)	
	Female	20 (50)	71 (52.2)	5 (29.4)	
	Unknown	1 (2.5)	17 (12.5)	1 (5.9)	
Reporting year [n (%)]	2004-2008	18 (45)	10 (7.4)	5 (29.4)	
	2009-2012	3 (7.5)	32 (23.5)	4 (23.5)	
	2013-2016	8 (20)	18 (13.2)	4 (23.5)	
	2017-2020	9 (22.5)	29 (21.3)	3 (17.7)	
	2021-2024 (June)	2 (5)	47 (34.6)	1 (5.9)	
Reporting top countries	USA	17 (42.5)	37 (27.2)	8 (47.1)	
	Others and unknown	23 (57.5)	99 (72.8)	9 (52.9)	

**Abbreviations:** DAPT: Dual antiplatelet therapy; and USA: The United States of America.

**Table 2:** Signal detection measures for the risk of arterial thrombo-embolic events with antiplatelet drugs.

**Drugs**	**PRR**	**Lower Limit 95% CI of PRR**	**Upper Limit 95% CI of PRR**	**RRR**	**χ2**	**Number of Reports**	**IC025**	**EBGM05**
**ASA-PPI Combination therapy**								
ASA/Lansoprazole	2.9	1.6	5	2.9	12.8	12	0.8	1.6
ASA/Omeprazole	1.6	0.8	3.1	1.6	1.1	8	0.3	0.8
ASA/Esomeprazole	6.1	3.1	11.7	6.1	29.4	8	1.3	2.9
ASA/Pantoprazole	2.1	1.2	3.7	2.1	6.1	12	0.6	1.2
**Clopidogrel-PPI Combination therapy**								
Clopidogrel/Lansoprazole	2.9	1.9	4.5	2.9	25.4	21	1	1.9
Clopidogrel/Omeprazole	6.3	4.6	8.5	6.3	160.9	37	1.9	4.5
Clopidogrel/Esomeprazole	6.1	3.8	9.6	6.1	63.7	16	1.6	3.6
Clopidogrel/Pantoprazole	3.7	2.6	5.3	3.7	55.4	29	1.3	2.5
Clopidogrel/Rabeprazole	30.9	24.3	39.1	30.9	939.7	33	3	18.9
**DAPT-PPI Combination therapy**								
ASA/Clopidogrel/Lansoprazole	5.6	2.5	13	5.6	15	5	1	2.2
ASA/Clopidogrel/Omeprazole	5.8	2.5	13.2	5.8	15.4	5	1	2.3
ASA/Clopidogrel/Pantoprazole	4	2	8.2	4	13.3	7	0.9	1.9

**Abbreviations:** ASA: Acetylsalicylic acid; PPI: Proton pump inhibitor; DAPT: Dual antiplatelet therapy; RRR: Relative reporting ratio; PRR: Proportional reporting ratio; χ2: Chi-square test statistics; IC: Information component; and EBGM: Empirical Bayes geometric mean.

**Table 3:** Interaction signal scores between antiplatelet (monotherapy and dual)-PPI combinations for the risk of arterial thrombo-embolic events.

**Drugs**	**EBGM**	**EB05**	**EB95**	**INTSS**
**ASA-PPI Combination therapy**				
ASA/Lansoprazole	2.9	1.8	4.6	NA
ASA/Omeprazole	1.5	0.9	2.8	
ASA/Esomeprazole	1.8	3.3	11.2	
ASA/Pantoprazole	2.1	1.3	3.4	
**Clopidogrel-PPI Combination therapy**				
Clopidogrel/Lansoprazole	2.9	2	4.2	NA
Clopidogrel/Omeprazole	6.3	4.7	8.3	
Clopidogrel/Esomeprazole	6.1	3.9	9.4	
Clopidogrel/Pantoprazole	3.7	2.7	5.1	
Clopidogrel/Rabeprazole	30.9	20.5	46.6	
**DAPT-PPI Combination therapy**				
ASA/Clopidogrel/Lansoprazole	5.6	2.6	12.2	0.57
ASA/Clopidogrel/Omeprazole	5.8	2.7	12.5	0.33
ASA/Clopidogrel/Pantoprazole	4	2.1	7.7	0.41

**Abbreviations:** ASA: Acetylsalicylic acid; PPI: Proton pump inhibitor; DAPT: Dual antiplatelet therapy; EBGM: Empirical Bayes geometric mean; EB05: Lower limit of 90% CI of EBGM; EB95: Upper limit of 90% CI of EBGM; INTSS: Interaction signal score; and NA: Not applicable.

## Data Availability

The datasets generated during and/or analyzed during the current study are publicly available from the USFDA AERS database (https://fis.fda.gov/sense/app/95239e26-e0be-42d9-a960-9a5f7f1c25ee/sheet/7a47a261-d58b-4203-a8aa-6d3021737452/state/analysis).

## LIST OF ABBREVIATIONS

DAPT: Dual Antiplatelet Therapy
PPIs: Proton Pump Inhibitors
ROR: Reporting Odds Ratio
