# Supplementary material for: Antiplatelet-Proton Pump Inhibitor Interactions and Arterial Thrombotic Events: A Pharmacovigilance Assessment using Disproportionality and Interaction Analysis
Source: Curr Cardiol Rev. 2025 May 12;21(6):e1573403X374906. doi: 10.2174/011573403X374906250417043907 (PMC12676020; doi:10.2174/011573403X374906250417043907)
Supplement: Supplementary file 1 [file CCR-21-6-E1573403X374906_SD1.pdf]

Supplementary Material

Antiplatelet-Proton Pump Inhibitor Interactions and Arterial Thrombotic Events: A Pharmacovigilance Assessment Using Disproportionality and Interaction Analysis

Kannan Sridharan[1,*]

[1]*Department of Pharmacology & Therapeutics, College of Medicine & Health Sciences, Arabian Gulf University, Manama, Kingdom of Bahrain*

Electronic Supplementary Table 1. List of Preferred Terms included in the Query.

Preferred Term	MedDRA code
Acute aortic syndrome	10074337
Acute coronary syndrome	10051592
Acute myocardial infarction	10000891
Amaurosis	10001902
Amaurosis fugax	10001903
Aneurysm thrombosis	10086560
Angioplasty	10002475
Angiostomy	10087774
Aortic aneurysm thrombosis	10087600
Aortic bypass	10057617
Aortic embolus	10002897
Aortic surgery	10061651
Aortic thrombosis	10002910
Aortogram abnormal	10057794
Arterectomy	10071026
Arterectomy with graft replacement	10003140
Arterial angioplasty	10081731
Arterial bypass operation	10056418
Arterial graft	10061655
Arterial occlusive disease	10062599
Arterial recanalisation procedure	10087576
Arterial revascularisation	10084482
Arterial stent insertion	10061657
Arterial therapeutic procedure	10052949
Arterial thrombosis	10003178
Arteriogram abnormal	10061659

Arteriogram carotid abnormal	10003195
Arteriotomy	10078636
Atherectomy	10063025
Atherosclerotic plaque rupture	10076604
Atrial appendage closure	10079735
Atrial appendage resection	10080843
Basal ganglia infarction	10069020
Basilar artery occlusion	10048963
Basilar artery thrombosis	10063093
Blindness transient	10005184
Brachiocephalic artery occlusion	10069694
Capsular warning syndrome	10067744
Carotid angioplasty	10071260
Carotid arterial embolus	10007684
Carotid artery bypass	10053003
Carotid artery occlusion	10048964
Carotid artery stent insertion	10066102
Carotid artery thrombosis	10007688
Carotid endarterectomy	10007692
Carotid revascularisation	10072559
Cerebellar artery occlusion	10053633
Cerebellar artery thrombosis	10008023
Cerebral angioplasty	10087440
Cerebral artery embolism	10008088
Cerebral artery occlusion	10008089
Cerebral artery stent insertion	10081893
Cerebral artery thrombosis	10008092
Cerebral bypass surgery	10089035
Cerebral hypoperfusion	10065384
Cerebral revascularisation	10071508
Cerebrovascular insufficiency	10058842
Cerebrovascular stenosis	10061751
Coeliac artery occlusion	10069696
Coronary angioplasty	10050329
Coronary arterial stent insertion	10052086
Coronary artery bypass	10011077
Coronary artery embolism	10011084
Coronary artery occlusion	10011086
Coronary artery reocclusion	10053261

Coronary artery surgery	10011090
Coronary artery thrombosis	10011091
Coronary endarterectomy	10011101
Coronary revascularisation	10049887
Coronary vascular graft occlusion	10075162
Embolia cutis medicamentosa	10058729
Embolism arterial	10014513
Endarterectomy	10014648
Femoral artery embolism	10068365
Hepatic artery embolism	10019635
Hepatic artery occlusion	10051991
Hepatic artery thrombosis	10019636
Hypothenar hammer syndrome	10063518
Iliac artery embolism	10021338
Iliac artery occlusion	10064601
Incomplete atrial appendage closure	10088942
Internal capsule infarction	10083408
Intra-aortic balloon placement	10052989
Intraoperative cerebral artery occlusion	10056382
Ischaemic cerebral infarction	10060840
Ischaemic stroke	10061256
Lacunar infarction	10051078
Left atrial appendage closure implant	10085044
Leriche syndrome	10024242
Mesenteric arterial occlusion	10027394
Mesenteric arteriosclerosis	10065560
Mesenteric artery embolism	10027395
Mesenteric artery stenosis	10027396
Mesenteric artery stent insertion	10071261
Mesenteric artery thrombosis	10027397
Metabolic stroke	10086596
Myocardial infarction	10028596
Myocardial necrosis	10028602
Ophthalmic artery occlusion	10086395
Ophthalmic artery thrombosis	10081144
Papillary muscle infarction	10033697
Penile artery occlusion	10068035
Percutaneous coronary intervention	10065608
Peripheral arterial occlusive disease	10062585

Peripheral arterial reocclusion	10069379
Peripheral artery angioplasty	10057518
Peripheral artery bypass	10072561
Peripheral artery occlusion	10057525
Peripheral artery stent insertion	10072562
Peripheral artery surgery	10082470
Peripheral artery thrombosis	10072564
Peripheral embolism	10061340
Peripheral endarterectomy	10072560
Popliteal artery entrapment syndrome	10071642
Post procedural myocardial infarction	10066592
Postinfarction angina	10058144
Precerebral artery embolism	10085250
Precerebral artery occlusion	10036511
Precerebral artery thrombosis	10074717
Profundaplasty	10078867
Pseudo-occlusion of internal carotid artery	10085779
Pulmonary angioplasty	10087828
Pulmonary artery occlusion	10078201
Pulmonary artery stent insertion	10088551
Pulmonary artery therapeutic procedure	10063731
Pulmonary artery thrombosis	10037340
Pulmonary endarterectomy	10072893
Pulmonary tumour thrombotic microangiopathy	10079988
Renal artery angioplasty	10057493
Renal artery occlusion	10048988
Renal artery revascularisation	10087816
Renal artery thrombosis	10038380
Renal embolism	10063544
Renal-limited thrombotic microangiopathy	10085346
Retinal artery embolism	10038826
Retinal artery occlusion	10038827
Retinal artery thrombosis	10038831
Segmental arterial mediolysis	10086467
Silent myocardial infarction	10049768
Spinal artery embolism	10049440
Spinal artery thrombosis	10071316
Splenic artery thrombosis	10074600
Splenic embolism	10068677

Stress cardiomyopathy	10066286
Subclavian artery embolism	10042332
Subclavian artery occlusion	10069695
Subclavian artery thrombosis	10042334
Thromboembolectomy	10064958
Thrombotic microangiopathy	10043645
Thrombotic thrombocytopenic purpura	10043648
Transient ischaemic attack	10044390
Truncus coeliacus thrombosis	10062363
Vascular pseudoaneurysm thrombosis	10078269
Vertebral artery occlusion	10048965
Vertebral artery thrombosis	10057777
Vertebrobasilar infarction	10089401
Visual acuity reduced transiently	10047532

Electronic Supplementary Table 2. Search strategy used in this study.

**Drug/Drug combinations**	**Search terms**
Acetylsalicylic acid with lansoprazole	**Drug:** Acetylsalicylic acid OR Aspirin AND lansoprazole AND NOT clopidogrel AND NOT warfarin AND NOT heparin AND NOT dabigatran AND NOT apixaban AND NOT rivaroxaban<br><br>**Role:** Primary suspect (for Acetylsalicylic acid and Aspirin)<br><br>**Adverse event SMQ (Narrow):** Embolic and Thrombotic Events, Arterial
Acetylsalicylic acid with dexlansoprazole	**Drug:** Acetylsalicylic acid OR Aspirin AND dexlansoprazole AND NOT clopidogrel AND NOT warfarin AND NOT heparin AND NOT dabigatran AND NOT apixaban AND NOT rivaroxaban<br><br>**Role:** Primary suspect (for Acetylsalicylic acid and Aspirin)<br><br>**Adverse event SMQ (Narrow):** Embolic and Thrombotic Events, Arterial
Acetylsalicylic acid with omeprazole	**Drug:** Acetylsalicylic acid OR Aspirin AND omeprazole AND NOT clopidogrel AND NOT warfarin AND NOT heparin AND NOT dabigatran AND NOT apixaban AND NOT rivaroxaban<br><br>**Role:** Primary suspect (for Acetylsalicylic acid and Aspirin)<br><br>**Adverse event SMQ (Narrow):** Embolic and Thrombotic Events, Arterial
Acetylsalicylic acid with esomeprazole	**Drug:** Acetylsalicylic acid OR Aspirin AND esomeprazole AND NOT clopidogrel AND NOT warfarin AND NOT heparin AND NOT dabigatran AND NOT apixaban AND NOT rivaroxaban<br><br>**Role:** Primary suspect (for Acetylsalicylic acid and Aspirin)<br><br>**Adverse event SMQ (Narrow):** Embolic and Thrombotic Events, Arterial
Acetylsalicylic acid with pantoprazole	**Drug:** Acetylsalicylic acid OR Aspirin AND pantoprazole AND NOT clopidogrel AND NOT warfarin AND NOT heparin AND NOT dabigatran AND NOT apixaban AND NOT rivaroxaban<br><br>**Role:** Primary suspect (for Acetylsalicylic acid and Aspirin)<br><br>**Adverse event SMQ (Narrow):** Embolic and Thrombotic Events, Arterial
Acetylsalicylic acid with rabepra-	**Drug:** Acetylsalicylic acid OR Aspirin AND rabeprazole AND NOT clopidogrel AND NOT warfarin AND NOT

zole	heparin AND NOT dabigatran AND NOT apixaban AND NOT rivaroxaban<br><br>**Role:** Primary suspect (for Acetylsalicylic acid and Aspirin)<br><br>**Adverse event SMQ (Narrow):** Embolic and Thrombotic Events, Arterial
Clopidogrel with lansoprazole	**Drug:** Clopidogrel AND lansoprazole AND NOT Acetylsalicylic acid AND NOT Aspirin AND NOT warfarin AND NOT heparin AND NOT dabigatran AND NOT apixaban AND NOT rivaroxaban<br><br>**Role:** Primary suspect (for clopidogrel)<br><br>**Adverse event SMQ (Narrow):** Embolic and Thrombotic Events, Arterial
Clopidogrel with dexlansoprazole	**Drug:** Clopidogrel AND dexlansoprazole AND NOT Acetylsalicylic acid AND NOT Aspirin AND NOT warfarin AND NOT heparin AND NOT dabigatran AND NOT apixaban AND NOT rivaroxaban<br><br>**Role:** Primary suspect (for clopidogrel)<br><br>**Adverse event SMQ (Narrow):** Embolic and Thrombotic Events, Arterial
Clopidogrel with omeprazole	**Drug:** Clopidogrel AND omeprazole AND NOT Acetylsalicylic acid AND NOT Aspirin AND NOT warfarin AND NOT heparin AND NOT dabigatran AND NOT apixaban AND NOT rivaroxaban<br><br>**Role:** Primary suspect (for clopidogrel)<br><br>**Adverse event SMQ (Narrow):** Embolic and Thrombotic Events, Arterial
Clopidogrel with esomeprazole	**Drug:** Clopidogrel AND esomeprazole AND NOT Acetylsalicylic acid AND NOT Aspirin AND NOT warfarin AND NOT heparin AND NOT dabigatran AND NOT apixaban AND NOT rivaroxaban<br><br>**Role:** Primary suspect (for clopidogrel)<br><br>**Adverse event SMQ (Narrow):** Embolic and Thrombotic Events, Arterial
Clopidogrel with pantoprazole	**Drug:** Clopidogrel AND pantoprazole AND NOT Acetylsalicylic acid AND NOT Aspirin AND NOT warfarin AND NOT heparin AND NOT dabigatran AND NOT apixaban AND NOT rivaroxaban<br><br>**Role:** Primary suspect (for clopidogrel)<br><br>**Adverse event SMQ (Narrow):** Embolic and Thrombotic Events, Arterial
Clopidogrel with rabeprazole	**Drug:** Clopidogrel AND rabeprazole AND NOT Acetylsalicylic acid AND NOT Aspirin AND NOT warfarin AND NOT heparin AND NOT dabigatran AND NOT apixaban AND NOT rivaroxaban<br><br>**Role:** Primary suspect (for clopidogrel)<br><br>**Adverse event SMQ (Narrow):** Embolic and Thrombotic Events, Arterial
Acetylsalicylic acid with clopidogrel with lansoprazole	**Drug:** Acetylsalicylic acid OR Aspirin AND Clopidogrel AND lansoprazole AND NOT warfarin AND NOT heparin AND NOT dabigatran AND NOT apixaban AND NOT rivaroxaban<br><br>**Adverse event SMQ (Narrow):** Embolic and Thrombotic Events, Arterial
Acetylsalicylic acid with clopidogrel with dexlansoprazole	**Drug:** Acetylsalicylic acid OR Aspirin AND Clopidogrel AND dexlansoprazole AND NOT warfarin AND NOT heparin AND NOT dabigatran AND NOT apixaban AND NOT rivaroxaban<br><br>**Adverse event SMQ (Narrow):** Embolic and Thrombotic Events, Arterial
Acetylsalicylic acid with clopidogrel with omeprazole	**Drug:** Acetylsalicylic acid OR Aspirin AND Clopidogrel AND omeprazole AND NOT warfarin AND NOT heparin AND NOT dabigatran AND NOT apixaban AND NOT rivaroxaban<br><br>**Adverse event SMQ (Narrow):** Embolic and Thrombotic Events, Arterial
Acetylsalicylic acid with clopidogrel with esomeprazole	**Drug:** Acetylsalicylic acid OR Aspirin AND Clopidogrel AND esomeprazole AND NOT warfarin AND NOT heparin AND NOT dabigatran AND NOT apixaban AND NOT rivaroxaban

	**Adverse event SMQ (Narrow):** Embolic and Thrombotic Events, Arterial
Acetylsalicylic acid with clopidogrel with pantoprazole	**Drug:** Acetylsalicylic acid OR Aspirin AND Clopidogrel AND pantoprazole AND NOT warfarin AND NOT heparin AND NOT dabigatran AND NOT apixaban AND NOT rivaroxaban<br><br>**Adverse event SMQ (Narrow):** Embolic and Thrombotic Events, Arterial
Acetylsalicylic acid with clopidogrel with rabeprazole	**Drug:** Acetylsalicylic acid OR Aspirin AND Clopidogrel AND rabeprazole AND NOT warfarin AND NOT heparin AND NOT dabigatran AND NOT apixaban AND NOT rivaroxaban<br><br>**Adverse event SMQ (Narrow):** Embolic and Thrombotic Events, Arterial
